# Prevalence and risk factors for the development of diabetes mellitus in Swedish cats

**DOI:** 10.1186/1751-0147-54-61

**Published:** 2012-10-31

**Authors:** Marie Sallander, Johanna Eliasson, Åke Hedhammar

**Affiliations:** 1 Department of Clinical Sciences, Swedish University of Agricultural Sciences, Box 7054, Uppsala, S-750 07, Sweden; 2 Department of Animal Environment and Health, Swedish University of Agricultural Sciences, Box 234, Skara, S-532 23, Sweden

**Keywords:** Cat, Diabetes, Diet, Activity, Obesity

## Abstract

**Background:**

The prevalence and risk factors for the development of feline diabetes mellitus (FDM) in Swedish cats have not previously been reported. The objective of the present pilot study was to indicate prevalence and possible risk factors for FDM in Swedish cats. Twenty diabetic cats from the database at the University Animal Hospital in Uppsala participated in the study, and these were matched with 20 healthy controls on sex and age. A mail-and-telephone questionnaire focusing on diet, activity and obesity was used.

**Results:**

The prevalence of FDM during the years 2000–2004 based on the results of the hospital records in the present study was 21 per 10,000 cats. The diabetic cats were on average 9 years old when the disease signs were discovered (median, min-max 2–15). Among FDM cases, it was more common to be male (n=17 males vs n=3 females; *P≤*0.05). Ten out of twenty owners to cases (50%) reported their cats to be obese at the time of the diagnosis (median 9 years, min-max 2–15), as compared to five out of twenty (25%) controls at the same age. The median BW at the time for diagnosis was 5.5 kg (min-max 2.0-9.0) for cases, and 5.0 kg (min-max 3.0-8.0 kg) for controls, respectively. Despite that both cases and controls had the same median age at the time of the study (13 years, min-max 3–18), a significantly higher number of controls were alive at that age (n=16 controls vs 8 cases; *P≤*0.05). A significantly higher proportion of cases that were obese at the time of the FDM diagnosis were dead at the time of the study compared to the proportion of controls that were obese at a similar age (*P≤*0.05).

The diets given at the time for diagnosis for cases compared to diet of the controls at a similar time were mainly commercial foods, and controls consumed a higher proportion of dry foods compared to cases (medians 79 vs 44% of DM intake/d, respectively; P*≤*0.05). Cases were less active compared to the controls (2.3 and 3.2 h/d, respectively; *P≤*0.05).

**Conclusions:**

The results indicate that the proportions of dry foods in the diet, to perform low activity and to be obese could be identified as preliminary risk factors for FDM in Swedish cats, and should be taken into account in preventive measures as well as in the design of future epidemiological studies in this population.

## Background

Today, feline diabetes mellitus (FDM) is one of the most frequently diagnosed endocrine disorders in cats, and cats commonly suffer from feline diabetes type 2 [1]. The prevalence of FDM has increased during many years, from approximately 8 to 124 per 10,000 cats between the years 1970 to 1999 at college veterinary hospitals in the US [2]. Two recent studies show estimations of the prevalence of FDM in different parts of the globe; 74 and 44 cases per 10,000 cats in Australia (two large feline-only clinics) and the UK (insured population), respectively [3,4].

FDM is influenced both by genetic and environmental factors. Burmese cats has been shown to be at a higher risk of developing FDM compared to non-pedigree cats [3,4], and in Australia the prevalence for this breed was 221 in 10,000 cats [3]. Other risk factors that have been shown to increase the risk of developing FDM are being male, neutered, old, inactive, kept indoors, obese or treated with drugs such as corticosteroids [1-6].

We have earlier evaluated the effects of diet, exercise, body-weight (BW) and obesity as risk factors in canine diabetes mellitus in Sweden [7]. Prevalence and risk factors for the development of diabetes mellitus (DM) in cats in Sweden have not previously been reported. Within the scope of our research on multi-factorial diseases in companion animals, the objective of the present pilot study was to indicate possible risk factors for FDM for further studies on molecular genetics and environmental exposure.

## Methods

### Selection of cases and controls

To be able to discover differences in dietary intake between individuals, we used the dietary intake to estimate the sample size. We supposed a range in consumption of food that was equal to 65 g dry matter /d (s=16), and the bounds of errors was <8 g of food intake (B=8). Because the range is often ~4 standard deviations, one-fourth of the range will provide an estimated value of the standard deviation. Given these assumptions and knowing that the total number of cats in the Swedish population is estimated to approximately 1.600.000, the minimum sample size was ~16 cases and 16 controls [8].

n=Nσ2N−1*B24+σ2=1,600,000*1621,600,000−1*824+162=16cats

From the clinical records at the university clinic (Swedish University of Agricultural Sciences, SLU), 34 cases diagnosed with FDM in the years 2000–2004 were found and selected for inclusion in the study. The diagnostic codes selected were FDM, FDM with ketoacidosis, FDM with complication, and FDM without complication. We checked the journal of each cat so that the selected cases did not have any other types of diseases which could be relevant to diabetes (ie acromegaly or pancreatitis), and that no cases were consuming medication that could give hyperglycaemic concentrations. The cases were born in 1986–2001, and chosen with no consideration taken to sex, age or breed. The follow-ups of the clinical records were made on average 2.7 years (min-max 0–6) after the clinical records time period.

When diagnosing FDM both clinical signs and diagnostic tests were considered. The diagnostic tests taken were a haematology-package (blood hemoglobin, blood hematokrit, blood leucocytes, white blood cell differential count), ALT (alanine aminotransferase), ALP (serum alkanine phosphatase), total thyroxine, calcium, phosphorous, sodium, potassium, cloride, magnesium, glucose and fructosamine. The normal glucose concentrations varied between 3–5 mmol/L, while individuals with FDM regularly had elevated glucose concentrations above 10 mmol/L. As cats might show falsely elevated glucose concentrations due to stress during the examination, the concentrations of fructosamine was also measured (normally 160–380 μmol/L, elevated if above 400–450 μmol/L). The fructosamine concentration gives a good indication of the blood glucose in the last three weeks [9]. The inclusion criteria were that the cat had at least one of the clinical signs typical for FDM such as polyuria, polydipsia, polyphagia, weight loss, gait, lethargy, vomiting, weakness, anorexia or coma. Also, the cat must have persistent fasting hyperglycemia (blood glucose concentration above 10 mmol/L) and fluctuating fructoseamine levels (above 400 μmol/L).

The controls (n=39) were selected from the same clinic as the cases, and were matched for sex (including neutering status) and year of birth. To find the control cats, the diagnostic codes used were: healthy, prophylaxis, veterinary examination, mechanical injury and fall from high altitude. The records of the controls were scrutinized so that they did not have any indication that the cats were not healthy. Before the interviews, the owners were asked if their cat was healthy or if it suffered from any disease (that would make them unsuitable to be a control). However, none of the controls were excluded for this reason.

### Collection of data and questionnaire

The owners of the cats were asked to participate in the study by a combined mail-and-telephone questionnaire (supplied if you send an e-mail to the main author). Within two weeks after the questionnaire was posted, a telephone interview based on the same questions was performed by a trained interviewer (the 2^nd^ author). After a letter including the questionnaire and subsequently at least five repeated phone-calls, the owners were deemed to be non-respondents.

The cases and controls were sent identical questionnaires, except for that the cases were also asked questions about their diabetes. The questionnaires included questions about demographics, diet, activity, BW and body-condition score (BCS) at different ages. Also, the cases were asked about signs shown before the FDM diagnosis, medical treatments and if the cats were given veterinary diets.

Demographics included questions on breed, year of birth, gender including neutered or intact status, and age of castration.

Feeding patterns included questions such as number of meals/d, commercial products used (processing type, frequency, amount), table foods (type, frequency, amount), treats and supplements (type, frequency, amount). All foods were recorded only if fed at least once per month. Treats were defined as some type of food given as a source of special delight, often given between meals. Supplements were defined as nutrients added to the diet, often to provide vitamins, minerals, fatty acids or amino acids.

Living patterns and activity included questions about how the cat was kept (indoors, outdoors, both), general activity (calm, moderately active, very active), physical activity (h/d), and presence of other cats in the household (yes/no, numbers).

The degree of obesity was estimated with a validated method with pictures of cats in different BCS’s [10]. Also, the BW at the time of the diagnosis was collected from the veterinary record of each individual. At the time of the study, cats were weighed by the owners. The owner held the cat, then receiving the weight of both the owner and the cat. After, the owner stood on the scale alone, and then the first sum was subtracted from the second sum to receive the body weight of the cat.

For cases, the age at the start of the FDM signs, the first signs noticed, how long time that passed from the first signs until the diagnosis was confirmed, treatment, and possible medical foods were also recorded.

### Data analysis

Summary statistics for all variables were calculated. Categorical variables were analysed with Fisher’s exact test and continuous variables with Mann–Whitney *U* test. For test of proportions, the 2-proportions test was used. For test of the number of male vs female cases that had been included in the study, the numbers were compared to if half of the males and half of the females would have been cases. A value of *P≤*0.05 was considered statistically significant. The data were analysed with Minitab [11].

## Results

### Response rate

The response rate among included cases and controls was 57 (20/34) and 51% (20/39), respectively. Reasons for no response were that the owners did not want to participate in a study, often because the cat was dead (11 cases, 8 controls), or that they could not be reached at their address/telephone (3 cases, 11 controls). Approximately a third (n=10/34) of the non-respondent cases had been euthanized when being diagnosed with FDM, while 20% of the non-respondent controls were dead for some reason (n=8/39).

The prevalence of FDM during the years 2000–2004 based on the results of the hospital records in the present study was 21 per 10,000 cats.

### Demographics

Among FDM cases (and by design consequently also among controls), it was more common to be male (n=17 males vs n=3 females; *P≤*0.05). Both cases and controls had the same median age at the time of the study (13 years, min-max 3–18). All cases were desexed at the median age of 12 months (min-max 7–24), and so were the controls (median 12 months, min-max 6–72). The breeds represented were European shorthair (19 cases, 20 controls) and Norwegian cat (1 case).

### Age at diagnosis and common signs for FDM

The diabetic cats were on average 9 years old when the disease signs were noted (median, min-max 2–15). The signs before or at the time for diagnosis were severe thirst (n=17), serious urination (n=15), loss of BW (n=12), extremely good appetite (n=8), apathy (n=5), loss of appetite (n=3), and stress (n=2). On average, the cats had signs in 2 months (median, min-max 3 days-2 years) before the diagnosis was established. One of the diabetic cats had a relative (sibling) that also had diabetes.

### Diet at the time of diagnosis for cases compared to diet of the controls at a similar age

At the time of the diagnosis of FDM, commercial foods were the main types of dietary intake given both to cases and controls; and controls consumed a higher proportion of dry foods compared to cases (medians 79 vs 44% of DM intake/d, respectively; P*≤*0.05; Table 1).

**Table 1:** Diet before/at FDM diagnosis for cases and controls at a similar age

**Food item**	**Cases (n=20)**			**Controls (n=20)**		
	**Proportion yes (%)**^**a**^	**Proportion (% of total intake, DM/d)**^**a, b**^		**Proportion yes (%)**^**a**^	**Proportion (% of total intake, DM/d)**^**a, b**^	
		**Median**	**Min-max**		**Median**	**Min-max**
Dry foods	85	44	0-100	85	79^*^	0-100
Canned foods	70	48	0-100	75	20	0-100
Table foods	65	10	5-30	80	6	1-20
Vitamins/minerals	40	-		25	-	
Treats	10	-		20	-	

^a^Fishers exact test significant at ^*^P≤0.05, ^**^P≤0.01, ^***^P≤0.001.

^b^To calculated the proportion of food given (g dry matter/d), the dry matter has been estimated to 90 and 20% in dry and canned/table foods, respectively.

At the time of the diagnosis of FDM, table foods constituted a minor part of the diets both for cases and controls. The few table foods fed were mainly animal products. At this time, supplements were given to cases and to controls to the same extent, and the most commonly fed type of products were vitamins, most commonly given on a daily-basis. The few cats that were given treats at the time of the diagnosis were served cat mint or an extra portion of dry foods in small amounts sporadically (Table 1).

### Living patterns and activity

Cases were less active compared to the controls (medians 2.3 and 3.2 h/d, respectively; *P≤*0.05; Table 2).

**Table 2:** General temperament of cases of FDM and controls as stated by the owners

	**Cases (n=20)**	**Controls (n=20)**
	**Proportion yes(%)**^**a**^	**Proportion yes (%)**^**a**^
>1 cat in household	80^b^	50
General temperament		
Calm	50	35
Moderately active	40	50
Very active	10	15
Activity, h/d		
<1	22	11
1-5	78	84
>5	0	5

^a^Fishers exact test significant at ^*^P≤0.05, ^**^P≤0.01, ^***^P≤0.001.

^b^*P*=0.096.

Most cats were kept both inside and outside (n=9 of the cases and n=13 of the controls, respectively). Fifty (n=10) and thirty (n=6) percent of the cases and controls, respectively, were only indoors, and only a few cats were only outdoors (medians 5 and 5% of the cases and controls, respectively).

Out of all cats in the study, a higher proportion was considered to be calm or moderately active compared to those considered very active by their owners. However, we found no significant difference between cases and controls in this matter (Table 2).

### Obesity

Ten out of twenty owners to cases (50%) reported their cats to be obese at the time of the diagnosis (median 9 years, min-max 2–15), as compared to five out of twenty (25%) controls at the same age. The median BW at the time for diagnosis was 5.5 kg (min-max 2.0-9.0) for cases, and 5.0 kg (min-max 3.0-8.0 kg) at a similar age for controls, respectively.

At the time of the study, n=17 of the cases and n=12 of the controls were regarded as obese, respectively. The median BW at the time for the study was 5.9 kg (min-max 2.0-9.0) for cases, and 5.0 kg (min-max 3.0-8.0 kg) for controls, respectively.

### Survival

At the time of the study (2.7 years after the diagnosis, min-max 0–6), 60% (n=12) of the diabetic cats were treated with some type of insulin (n=6 obese, n=6 non-obese), while 40% had been put to sleep (n=3 obese, n=5 non-obese). The types of insulin used were commonly Caninsulin, Insuvet or Minidiab. Out of the cats originally put on insulin treatment, only one had been put down after a non-successful compliance.

At the time of the study, a higher proportion of controls were alive compared to the cases (n=8 of the cases vs n=16 of the controls; *P≤*0.05). A significantly higher proportion of cases that were obese at the time of the FDM diagnosis were dead at the time of the study compared to the proportion of controls that were obese at a similar age (*P≤*0.05; Table 3).

**Table 3:** Proportion obese cats at FDM diagnosis, and at the time of the study

**The cat was obese**		**Proportion (%)**^**a**^	
**at the time of the FDM diagnosis**	**at the time of the study**	**Cases**	**Controls**
No	No	10	10
No	Yes	20	50
Yes	No	5	0
Yes	Yes	5	20
No	Diseased	25	15
Yes	Diseased	35	5^*^

^a^Fishers exact test significant at ^*^P≤0.05, ^**^P≤0.01, ^***^P≤0.001.

Proportion diseased at the time of the study.

## Discussion

The present study used a combined mail and telephone questionnaire. The way of collecting data was chosen as it has been shown to be efficient in previous studies in the Swedish dog population [12-14]. By both sending the owner a questionnaire and then making a phone-call to the owners, the owners were given time to collect the data needed for the questionnaire, but also had the chance to get answers from the interviewer if they did not understand a question. This possibly made the questionnaires more complete compared to a simpler posted questionnaire.

When performing the present retrospective study, we used a university clinic register for patients who had been at the clinic at some time in the past. Of course, for example the data of current addresses, telephone numbers, whether owners or cats were alive was not always updated. This is always a dilemma when making a retrospective study, and might be one reason why the present study had a lower response frequency than the previous studies by the same research group. Also, the memory of the owners might be hampered as some years have passed since the cats were young, since they were diagnosed with diabetes. However, in previous studies in humans (cancer study which uses a previously validated survey) and in companion animals (survey of diet and exercise in dogs that uses a previously validated questionnaire), validated dietary questionnaires has shown that it is indeed possible to get data from the whole life-span with quite a high amount of certainty [15,16]. However, none of these studies are made in cats, and basic and larger studies would be needed in cats.

Due to relatively few numbers of cases leading to low power, we probably failed to get some significant data (for example indoor/outdoor status of cases and controls). Therefore, we look at the present report as a pilot study in the Swedish cat population.

In the present study all female and male cats recruited were castrated, and there were a higher proportion of cases that were males compared to females. This is in accordance with earlier studies [17].

In a recent Dutch study, the energy percentage from dry foods was not significantly correlated with the development of FDM of cats [6]. In the present study, we were not able to compare the energy percentages, but we found that at the time of the diagnosis of the FDM (and at the same age for controls), the diabetic cats consumed a significantly lower proportion of dry foods (and higher proportion of canned foods) compare to controls (% of DM intake/d). Canned foods are commonly more energy-dense due to higher concentrations of fat content compared to dry foods (DM-basis), indicating that diabetic cats are more commonly given energy dense diets compared to controls. Canned food often smells and taste very good, which makes it easy to over-feed, which might lead to obesity and related diseases such as diabetes.

Today, a diet with a high proportion of protein and low proportions of fat and carbohydrates are often recommended both for weight loss and for diabetes in cats. Suitable diets for that purpose are often canned diets, but there are also dry veterinary diets that fulfil this purpose. From this study we could not see any pattern that veterinarians recommended that the cats should switch to a canned diet after the diabetes diagnosis. Instead, the recommendation was first based on what disease the cats suffered from, and then the type of diet (dry or canned) and brand was chosen depending on what the cat was used to from before the diagnosis.

In Sweden, cats are not commonly exercised on lead or on walks. Outdoor cats are running around freely, and it is difficult to know the exact amount and frequency of exercise performed. Therefore, it is important to emphasize that the numbers given for activity in cats are estimations made by the owners.

In a recent Dutch study, the researchers found a significant difference between cases and controls in the frequency of FDM between those cats that had been indoors compared to those that had been outdoors [6]. In the present study, we did not note the same difference. Neither did we find any difference whether the cases or controls were obese or not depending on if they were solely kept indoors or not. Most Swedish cats are allowed to be partly outside, and the numbers of cats kept only inside was low. Possibly, this might be a reason for that we did not find a significant difference between the groups.

The study mentioned above also reported that a low physical activity was significantly correlated with the development of FDM (*P*=0.004), which is in correspondence with our study [6]. We noted that the control cats were in activity longer each day compared to the cats with diabetes.

In dogs, we have previously shown that owners tend to under-estimate the BCS in their pet. Therefore, we should interpret the results of the BCS with some caution.

A significantly higher proportion of cases that were obese at the time of the FDM diagnosis were dead at the time of the study compared to the proportion of controls that were obese at a similar age. This difference might slightly be due to that among non-respondents, approximately a third of the cases had chosen to put their cats to sleep when diagnosed with FDM, while only 20% of the controls did not participate due to that the cat was diseased.

At the time of the study, a higher proportion of controls were alive compared to the cases. This indicates that obesity not only is a risk factor for FDM, but also something that may shorten the total life-time of the cats.

The cases had had the present BCS for a shorter time than the controls, indicating that they probably had decreased in BCS during some time. Possibly, the diabetic cats either did decrease in BCS due to the disease, or had been told to decrease in BW by the veterinarians as this is known to be a risk factor for FDM [5].

## Conclusions

The present study has indicated that in Sweden, FDM has a prevalence of 21 in 10,000 cats. The results indicate that the proportions of dry foods in the diet, to perform low activity and to be obese could be identified as preliminary risk factors for FDM in the Swedish cat population, and should be taken into account in preventive measures as well as in the design of future epidemiological studies in this population.

## Competing interests

None of the authors of this paper has a financial or personal relationship with other people or organizations that could inappropriately influence or bias the content of the paper.

## Authors’ contributions

MS, JE and ÅH have been involved in the initial design of the study. JE has been responsible for performing the combined mail-and telephone interviews. MS has been the main responsible and ÅH has been supporting in the data analysis. All authors have contributed to the editing of the manuscript. All read and approved the final manuscript.
